# Heavy metals analysis and quality assessment in drinking water – Khorramabad city, Iran

**DOI:** 10.1016/j.dib.2017.11.078

**Published:** 2017-12-08

**Authors:** Mansour Ghaderpoori, Bahram kamarehie, Ali Jafari, Afshin Ghaderpoury, Mohammadamin Karami

**Affiliations:** a Nutritional Health Research Center, Lorestan University of Medical Sciences, Khorramabad, Iran; b Department of Environmental Health Engineering, School of Health and Nutrition, Lorestan University of Medical Sciences, Khorramabad, Iran; c Students Research Committee, Shahid Beheshti University of Medical Sciences, Tehran, Iran

**Keywords:** Drinking water quality, Heavy metals, Monitoring, Khorramabad city

## Abstract

Continuous monitoring of drinking water quality is essential in terms of heavy metals and toxic substances. The general objective of this study were to determine the concentration of heavy metals in drinking water of Khorramabad city and to determine the water quality indices (The heavy metal pollution index and heavy metal evaluation index). According to the city map, 45 points were selected for drinking water sampling through the city distribution system. The results of this study showed that the average concentration of heavy metals such as Zn, Pb, Cd, Cr, and Cu were 47.01 μg/l, 3.2 μg/l, 0.42 μg/l, 5.08 μg/l, and 6.79 μg/l, respectively. The HPI and HEI (water quality indices) for Zn, Pb, Cd, Cr, and Cu were 46.58, 46.58, respectively. According to the indices, the city drinking water quality is good in terms of heavy metals.

**Specifications Table**

**Table:** 

Subject area	*Chemistry, biology*
More specific subject area	*Water monitoring and quality*
Type of data	*Table, figure*
How data was acquired	*ICP-OES (Instrument Model: Varian VISTA-MPX)*
Data format	*Raw, analyzed,*
Experimental factors	*Measuring the concentration of heavy metals (Zn, Pb, Cd, Cr, and Cu) in the samples of drinking water distribution system. After determining the concentration, water quality pollution indices were calculated.*
Experimental features	*According to the city map, 45 points of drinking water in distribution system were selected as sampling point. Until concentration measurement, all samples were stored in standard conditions and were analyzed for heavy metals*
Data source location	*Khorramabad city Iran (33° 48′ N, 48° 35′ E)., Lorestan province, west of Iran*
Data accessibility	*Data are included in this article and supplemented excel file*

**Value of the Data**

• There is always the possibility of contamination of water resources with heavy metals and toxic substances, therefore, continuous monitoring is essential.
• Heavy metals can accumulate in human body and other living organisms over a long period and may cause adverse effects on human health.
• The main sources of heavy metals contamination in drinking water including heavy metals leakage through iron pipes in distribution systems and due to geological contamination of the region that water originates from.
• One of the most important methods for drinking water quality determination is the measurement of heavy metal pollution by indices, so, heavy metals data can be used for determination of the water quality indices determination.

## 1 Location data

**Fig. 1:**
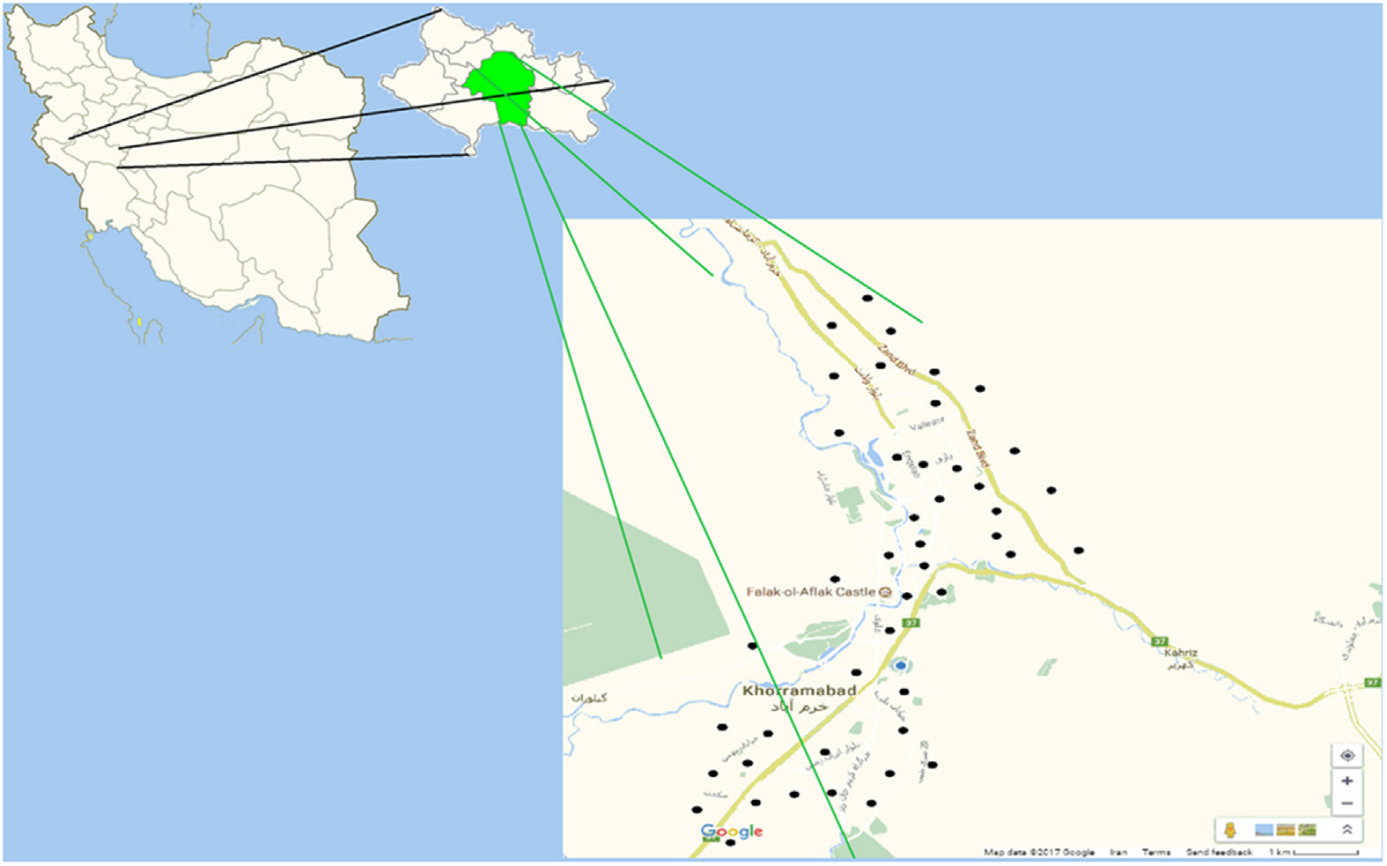
Location and sampling points in Khorramabad city, Iran.

Khorramabad, one of the cities of Lorestan province, located at west of Iran (33° 48′ N, 48° 35′ E). Fig. 1 shows the location of the city and the sampling points the study area. The city area is about 6233 km^2^. Based on the latest population census in Iran (2016), its population was 506,471 persons.

## 2 Experimental design, materials, and methods

In this study, 45 stations were selected as sampling points in a way that covers the whole city distribution system. Sampling was performed according to a standards procedure. The collected samples were also kept in accordance with standard methods for water and wastewater. Further, ICP-OES (Instrument Model: Varian VISTA-MPX) was used to measure the concentrations of heavy metals. Table 1 shows the concentration of measured heavy metals.

**Table 1:** The concentrations of heavy metals in the study area.

**Station**	**1**	**2**	**3**	**4**	**5**	**6**	**7**	**8**	**9**	**10**	**11**	**12**	**13**	**14**	**15**	**16**	**17**	**18**	**19**	**20**	**21**	**22**	**23**
**Zn**	33.40	43.30	56.21	44.50	78.90	43.46	103.61	59.29	56.94	93.11	64.58	79.27	45.57	54.48	54.48	98.27	61.54	30.01	8.32	7.41	8.72	28.25	13.35
**Pb**	0.67	1.02	0.58	0.59	1.32	3.06	4.74	3.64	1.35	4.54	5.95	2.83	6.57	0.35	0.35	2.29	2.59	3.43	6.38	6.00	4.49	8.27	3.50
**Cd**	0.05	0.05	0.02	–	–	0.13	0.12	0.14	0.11	0.13	0.08	0.42	0.04	0.13	0.13	0.10	0.62	0.94	1.49	1.44	0.85	0.52	0.73
**Cr**	3.04	8.14	10.39	7.13	7.62	3.39	4.75	4.87	4.79	6.95	7.87	6.39	4.31	7.57	7.57	7.80	6.39	0.39	3.42	2.86	5.32	2.03	0.60
**Cu**	0.17	0.10	3.15	0.36	0.44	10.87	11.48	5.61	6.01	39.31	3.59	6.01	9.44	5.39	5.39	9.35	2.59	3.36	4.06	5.26	3.45	3.30	3.26
**Station**	**24**	**25**	**26**	**27**	**28**	**29**	**30**	**31**	**32**	**33**	**34**	**35**	**36**	**37**	**38**	**39**	**40**	**41**	**42**	**43**	**44**	**45**	
**Zn**	70.88	87.11	94.23	104.77	69.04	69.04	81.40	11.58	9.88	9.81	12.80	12.88	21.80	18.85	19.31	17.50	20.28	46.417	54.712	51.103	37.65	27.221	
**Pb**	1.00	4.87	4.33	5.01	4.41	4.41	0.73	4.39	3.50	6.39	2.94	2.83	3.09	3.08	1.65	1.19	3.11	2.11	2.05	1.93	3.53	2.73	
**Cd**	0.15	0.10	0.22	0.14	0.56	0.56	0.44	1.00	0.92		0.64	0.66	0.75	0.80	0.35	0.36	0.69	0.328	0.608	0.154	0.56	0.122	
**Cr**	5.01	8.19	6.17	6.69	5.82	5.82	1.86	4.42	0.64	3.23	5.54	3.55	10.76	5.62	3.71	3.50	5.68	3.35	1.24	1.16	7.63	5.32	
**Cu**	16.32	17.62	21.36	21.37	9.15	9.15	15.04	3.88	4.04	3.43	4.21	3.22	3.70	4.22	3.22	3.23	3.21	3.18	3.44	3.35	3.26	3.84	

After determining the concentration, water quality pollution indices were calculated. The heavy metal pollution index (HPI) indicates the overall quality of the drinking water in terms of heavy metals [1], [2], [3], [4], [5], [6], [7]. This index is calculated according to Eqs. (1), (2) as follows:

(1) $$HPI=\frac{\sum_{i=1}^{n}W_{i}.Q_{i}}{\sum_{i=1}^{n}W_{i}}$$

(2) $$Q_{i}=\sum_{i=1}^{n}\frac{M_{i}(-)I_{i}}{S_{i}-I_{i}}\times 100$$

where, *Q*_i_ and *W*_i_ are the sub-index of the *i*th parameter and the unit weightage of the *i*th parameter, respectively. n is the number of parameters considered. *M*_i_, *I*_i_, and *S*_i_ are the monitored values of heavy metal, ideal and standard values of the *i*th parameter, respectively. The sign (−) indicates the numerical differences of the two values, ignoring the algebraic sign. Water quality based on HPI can be classified into three categories including: low (less than 100), the threshold risk (equal to 100), and high (more than 100). If HPI is more than 100, water cannot be used for drinking. Measured values of HPI index for the drinking water samples are presented in Table 2. Heavy metal evaluation index (HEI) is a method of estimating the water quality with focus on heavy metals in drinking water [4], [6], [7], [8], [9], [10]. This index is calculated according to Eq. (3), as follows:

(3) $$HEI=\sum_{i=1}^{n}\frac{H_{c}}{H_{mac}}$$

where, *H*_c_ and *H*_mac_ are the monitored values and maximum admissible concentration of the *i*th parameter, respectively. The classifications of the HEI index is as follows: low (less than 10), medium (between 10 and 20), and high (more than 20). Table 2, Table 3 show the used constants and the values of the calculated HPI and HEI, respectively [11], [12], [13], [14].

**Table 2:** Applied parameters and constants for calculation of HPI and HPI (according to WHO guidelines).

**Metal**	MCL* (µg/l)	***W***_**i**_	***I***_**i**_	***S***_**i**_	***H***_**max**_
**As**	50	0.02	10	50	50
**Zn**	5000	0.0002	3000	5000	5000
**Pb**	1.5	0.7	10	100	1.5
**Cd**	3	0.3	3	5	3
**Cr**	50	0.02	50	1	50
**Cu**	1000	0.001	2000	1000	1000
**Mn**	50	0.02	500	100	50

**Table 3:** The calculated indices, HPI and HEI, at the sampling points for heavy metals.

**Station**	**Zn**	**Pb**	**Cd**	**Cr**	**Cu**	**C**_**d**_	**Qi-Zn**	**Qi-Pb**	**Qi-Cd**	**Qi-Cr**	**Qi-Cu**	**Qi-Mn**	ΣWi*Qi	**HPI**	**HPI classification**	**HEI**	**HEI classification**
**1**	33.4	0.67	0.046	3.04	0.17	−5.52	148.33	10.37	147.70	95.84	199.98	125.00	56.71	53.44238	Low heavy metals	0.52965	Low heavy metals
**2**	43.3	1.02	0.046	8.14	0.1	−3.58	147.84	9.98	147.70	85.43	199.99	125.00	56.23	52.98961	Low heavy metals	0.866893	Low heavy metals
**3**	56.21	0.58	0.023	10.39	3.15	−3.13	147.19	10.47	148.85	80.84	199.69	125.00	56.83	53.55025	Low heavy metals	0.616525	Low heavy metals
**4**	44.5	0.59	0	7.13	0.36	−4.22	147.78	10.46	150.00	87.49	199.96	125.00	57.30	53.99378	Low heavy metals	0.545193	Low heavy metals
**5**	78.9	1.32	0.001	7.62	0.44	−3.56	146.06	9.64	149.95	86.49	199.96	125.00	56.70	53.42544	Low heavy metals	1.048953	Low heavy metals
**6**	43.46	3.06	0.127	3.39	10.87	−3.77	147.83	7.71	143.65	95.12	198.91	125.00	53.62	50.5312	Low heavy metals	2.169695	Low heavy metals
**7**	103.61	4.74	0.124	4.75	11.48	−2.18	144.82	5.84	143.80	92.35	198.85	125.00	52.31	49.28936	Low heavy metals	3.328535	Low heavy metals
**8**	59.29	3.64	0.137	4.87	5.61	−2.89	147.04	7.07	143.15	92.10	199.44	125.00	52.96	49.90817	Low heavy metals	2.587201	Low heavy metals
**9**	56.94	1.35	0.109	4.79	6.01	−4.45	147.15	9.61	144.55	92.27	199.40	125.00	55.17	51.98541	Low heavy metals	1.049531	Low heavy metals
**10**	93.11	4.54	0.127	6.95	39.31	−1.56	145.34	6.07	143.65	87.86	196.07	125.00	52.32	49.3064	Low heavy metals	3.265932	Low heavy metals
**11**	64.58	5.95	0.084	7.87	3.59	−0.37	146.77	4.50	145.80	85.98	199.64	125.00	51.84	48.84903	Low heavy metals	4.168573	Low heavy metals
**12**	79.27	2.83	0.423	6.39	6.01	−2.82	146.04	7.97	128.85	89.00	199.40	125.00	49.24	46.40056	Low heavy metals	2.177331	Low heavy metals
**13**	45.57	6.57	0.042	4.31	9.44	−1.15	147.72	3.81	147.90	93.24	199.06	125.00	52.13	49.12484	Low heavy metals	4.498754	Low heavy metals
**14**	54.48	0.35	0.133	7.57	5.39	−4.18	147.28	10.72	143.35	86.59	199.46	125.00	55.47	52.27225	Low heavy metals	0.445353	Low heavy metals
**15**	54.48	0.35	0.133	7.57	5.39	−4.18	147.28	10.72	143.35	86.59	199.46	125.00	55.47	52.27225	Low heavy metals	0.445353	Low heavy metals
**16**	98.27	2.29	0.101	7.8	9.35	−2.81	145.09	8.57	144.95	86.12	199.07	125.00	54.43	51.29306	Low heavy metals	1.745337	Low heavy metals
**17**	61.54	2.59	0.616	6.39	2.59	−2.92	146.92	8.23	119.20	89.00	199.74	125.00	46.53	43.84891	Low heavy metals	2.074698	Low heavy metals
**18**	30.014	3.43	0.942	0.39	3.36	−4.26	148.50	7.30	102.90	101.24	199.66	125.00	41.23	38.85626	Low heavy metals	2.617829	Low heavy metals
**19**	8.315	6.38	1.486	3.42	4.06	−1.11	149.58	4.02	75.70	95.06	199.59	125.00	30.66	28.88833	Low heavy metals	4.82279	Low heavy metals
**20**	7.408	6	1.443	2.86	5.26	−1.56	149.63	4.44	77.85	96.20	199.47	125.00	31.62	29.79607	Low heavy metals	4.544942	Low heavy metals
**21**	8.717	4.49	0.847	5.32	3.45	−1.95	149.56	6.12	107.65	91.18	199.66	125.00	41.63	39.23275	Low heavy metals	3.38726	Low heavy metals
**22**	28.253	8.27	0.521	2.03	3.3	−0.63	148.59	1.92	123.95	97.90	199.67	125.00	43.72	41.19667	Low heavy metals	5.736551	Low heavy metals
**23**	13.354	3.5	0.726	0.6	3.26	−4.22	149.33	7.22	113.70	100.82	199.67	125.00	44.41	41.85019	Low heavy metals	2.593264	Low heavy metals
**24**	70.88	1	0.147	5.01	16.32	−4.58	146.46	10.00	142.65	91.82	198.37	125.00	54.86	51.69524	Low heavy metals	0.846363	Low heavy metals
**25**	87.11	4.87	0.104	8.19	17.62	−0.95	145.64	5.70	144.80	85.33	198.24	125.00	52.36	49.34404	Low heavy metals	3.480175	Low heavy metals
**26**	94.23	4.33	0.219	6.17	21.36	−1.94	145.29	6.30	139.05	89.45	197.86	125.00	51.14	48.19158	Low heavy metals	3.123273	Low heavy metals
**27**	104.77	5.01	0.142	6.69	21.37	−1.34	144.76	5.54	142.90	88.39	197.86	125.00	51.75	48.76148	Low heavy metals	3.563457	Low heavy metals
**28**	69.043	4.41	0.563	5.82	9.15	−1.91	146.55	6.21	121.85	90.16	199.09	125.00	45.93	43.28537	Low heavy metals	3.267025	Low heavy metals
**29**	69.043	4.41	0.563	5.82	9.15	−1.91	146.55	6.21	121.85	90.16	199.09	125.00	45.93	43.28537	Low heavy metals	3.267025	Low heavy metals
**30**	81.398	0.73	0.435	1.86	15.04	−5.72	145.93	10.30	128.25	98.24	198.50	125.00	50.88	47.94344	Low heavy metals	0.700186	Low heavy metals
**31**	11.579	4.39	1	4.42	3.88	−2.26	149.42	6.23	100.00	93.02	199.61	125.00	39.45	37.17795	Low heavy metals	3.354596	Low heavy metals
**32**	9.882	3.5	0.92	0.64	4.04	−4.14	149.51	7.22	104.00	100.73	199.60	125.00	41.50	39.10643	Low heavy metals	2.658816	Low heavy metals
**33**	9.814	6.39		3.23	3.43	−1.66	149.51	4.01	150.00	95.45	199.66	125.00	52.95	49.89287	Low heavy metals	4.329993	Low heavy metals
**34**	12.804	2.94	0.642	5.54	4.21	−2.97	149.36	7.84	117.90	90.73	199.58	125.00	45.91	43.25787	Low heavy metals	2.291571	Low heavy metals
**35**	12.884	2.83	0.655	3.55	3.22	−3.71	149.36	7.97	117.25	94.80	199.68	125.00	45.88	43.23137	Low heavy metals	2.181797	Low heavy metals
**36**	21.799	3.09	0.748	10.76	3.7	−1.10	148.91	7.68	112.60	80.08	199.63	125.00	43.99	41.44882	Low heavy metals	2.532593	Low heavy metals
**37**	18.846	3.08	0.803	5.62	4.22	−2.80	149.06	7.69	109.85	90.57	199.58	125.00	43.38	40.8764	Low heavy metals	2.441389	Low heavy metals
**38**	19.308	1.65	0.346	3.71	3.22	−4.54	149.03	9.28	132.70	94.47	199.68	125.00	51.42	48.45771	Low heavy metals	1.296615	Low heavy metals
**39**	17.503	1.19	0.361	3.5	3.23	−4.91	149.12	9.79	131.95	94.90	199.68	125.00	51.56	48.59092	Low heavy metals	0.990397	Low heavy metals
**40**	20.275	3.11	0.686	5.68	3.21	−2.80	148.99	7.66	115.70	90.45	199.68	125.00	45.11	42.50598	Low heavy metals	2.422865	Low heavy metals
**41**	46.417	2.11	0.328	3.35	3.18	−4.35	147.68	8.77	133.60	95.20	199.68	125.00	51.35	48.38858	Low heavy metals	1.595463	Low heavy metals
**42**	54.712	2.05	0.608	1.24	3.44	−5.00	147.26	8.83	119.60	99.51	199.66	125.00	47.28	44.55583	Low heavy metals	1.608516	Low heavy metals
**43**	51.103	1.93	0.154	1.16	3.35	−5.26	147.44	8.97	142.30	99.67	199.67	125.00	54.19	51.06416	Low heavy metals	1.374771	Low heavy metals
**44**	37.65	3.53	0.56	7.63	3.26	−1.91	148.12	7.19	122.00	86.47	199.67	125.00	46.59	43.90398	Low heavy metals	2.70339	Low heavy metals
**45**	27.221	2.73	0.122	5.32	3.84	−3.36	148.64	8.08	143.90	91.18	199.62	125.00	53.88	50.77032	Low heavy metals	1.976351	Low heavy metals
**Mean**	**47.01**	**3.20**	**0.42**	**5.08**	**6.79**	**−3.11**	**147.70**	**7.64**	**130.06**	**91.90**	**199.34**	**125.00**	**49.43**	**46.58**	–	**2.33**	–
