# Supplementary material for: Heavy metals analysis and quality assessment in drinking water – Khorramabad city, Iran
Source: Data Brief. 2017 Dec 8;16:685–92. doi: 10.1016/j.dib.2017.11.078 (PMC5847489; doi:10.1016/j.dib.2017.11.078)
Supplement: Supplementary file 1 — Supplementary material [file mmc1.docx]

Dear Professor,

Editor of **Data in Brief**

The authors of this article declare that they have no conflict of interests.

Mansour Ghaderpoori, Bahram kamarehie, Ali Jafari, Afshin Ghaderpoury, Mohammadamin Karami
